# Diagnosis of Alzheimer’s Disease via Multi-Modality 3D Convolutional Neural Network

**DOI:** 10.3389/fnins.2019.00509

**Published:** 2019-05-31

**Authors:** Yechong Huang, Jiahang Xu, Yuncheng Zhou, Tong Tong, Xiahai Zhuang, the Alzheimer’s Disease Neuroimaging Initiative (ADNI)

**Affiliations:** ^1^School of Data Science, Fudan University, Shanghai, China; ^2^Fujian Provincial Key Laboratory of Medical Instrument and Pharmaceutical Technology, Fuzhou, China

**Keywords:** Alzheimer’s disease, multi-modality, image classification, CNN, deep learning, hippocampal

## Abstract

Alzheimer’s disease (AD) is one of the most common neurodegenerative diseases. In the last decade, studies on AD diagnosis has attached great significance to artificial intelligence-based diagnostic algorithms. Among the diverse modalities of imaging data, T1-weighted MR and FDG-PET are widely used for this task. In this paper, we propose a convolutional neural network (CNN) to integrate all the multi-modality information included in both T1-MR and FDG-PET images of the hippocampal area, for the diagnosis of AD. Different from the traditional machine learning algorithms, this method does not require manually extracted features, instead, it utilizes 3D image-processing CNNs to learn features for the diagnosis or prognosis of AD. To test the performance of the proposed network, we trained the classifier with paired T1-MR and FDG-PET images in the ADNI datasets, including 731 cognitively unimpaired (labeled as CN) subjects, 647 subjects with AD, 441 subjects with stable mild cognitive impairment (sMCI) and 326 subjects with progressive mild cognitive impairment (pMCI). We obtained higher accuracies of 90.10% for CN vs. AD task, 87.46% for CN vs. pMCI task, and 76.90% for sMCI vs. pMCI task. The proposed framework yields a state-of-the-art performance. Finally, the results have demonstrated that (1) segmentation is not a prerequisite when using a CNN for the classification, (2) the combination of two modality imaging data generates better results.

## Introduction

Aging of the global population results in an increasing number of people with dementia. Recent studies indicate that 50 million people are living with dementia (Patterson, 2018), of whom 60–70% have Alzheimer’s Disease (AD) (World Health Organization, 2012). Known as one of the most common neurodegenerative diseases, AD can result in severe cognitive impairment and behavioral issues.

Mild cognitive impairment (MCI) is a neurological disorder, which may occur as a transitional stage between normal aging and the preclinical phase of dementia. MCI causes cognitive impairments with a minimal impact on instrumental activities of daily life (Petersen et al., 1999, 2018). MCI is a heterogeneous group and can be classified according to its various clinical outcomes (Huang et al., 2003). In this work, we partitioned MCI into progressive MCI (pMCI) and stable MCI (sMCI), which are retrospective diagnostic terms based on the clinical follow-up according to the DSM-5 criteria (American Psychiatric Association, 2013). The term pMCI, refers to MCI patients who develop dementia in a 36-month follow-up, while sMCI is assigned to MCI patients when they do not convert. Distinguishing between pMCI and sMCI plays an important role in the early diagnosis of dementia, which can assist clinicians in proposing effective therapeutic interventions for the disease process (Samper-González et al., 2018).

With the progression of MCI and AD, the structure and metabolic rate of the brain changes accordingly. The phenotypes include the shrinkage of cerebral cortices and hippocampi, the enlargement of ventricles, and the change of regional glucose uptake. These changes could be quantified with the help of medical imaging techniques such as magnetic resonance (MR) and positron-emission tomography (PET) (Correa et al., 2009). For instance, T1-weighted magnetic resonance image (T1-MRI) provides high-resolution information for the brain structure, making it possible to accurately measure structural metrics like thickness, volume and shape. Meanwhile, 18-Fluoro-DeoxyGlucose PET (^18^F-FDG-PET or FDG-PET) indicates the regional cerebral metabolic rate of glucose, making it possible to evaluate the metabolic activity of the tissues. Other tracers, such as ^11^C-PiB and ^18^F-THK, are also widely used in AD diagnosis (Jack et al., 2008b; Harada et al., 2013), as they are sensitive to the pathology of AD as well. By analyzing these medical images, one can obtain important references to assist the diagnosis and prediction of AD (Desikan et al., 2009).

This work aims at distinguishing AD or potential AD patients from cognitively unimpaired (labeled as CN) subjects accurately and automatically using medical images of the hippocampal area and recent techniques in deep learning, as it facilitates a fast-preclinical diagnosis. The method is further extended for the classification between sMCI and pMCI so that an early diagnosis of dementia would be possible. Data of two modalities were used. i.e., the T1-MRI and ^18^F-FDG-PET, as they provide complementary information.

Numerous studies have been published on diagnosing AD by utilizing these two methods. Using T1-MRI, Sorensen et al. segmented the brains and extracted features of thickness and volumetry in the selected regions of interest (ROIs) (Sorensen et al., 2017). A linear discriminant analysis (LDA) was used to classify AD, MCI, and CN. David et al. implemented the kernel metric learning method in the classification (Cárdenas-Peña et al., 2017). Another popular machine learning method is the random forest. Lebedeva et al. (2017) extracted the structural features of MRI and used mini-mental state examination (MMSE) as a cognitive measure. Ardekani et al. (2017) took the hippocampal volumetric integrity of MRI and neuropsychological scores as the selected features. Both studies used the random forest. As for ^18^F-FDG-PET, Silveira and Marques (2010) proposed a boosting learning method that used a mixture of simple classifiers to perform voxel-wise feature selections. Cabral and Silveira (2013) used favorite class ensembles to form ensembled support vector machine (SVM) and random forest.

In addition to the single modality classifications, taking both T1-MRI and ^18^F-FDG-PET into consideration is also a major concern for research on AD diagnosis. Gray et al. (2013) took regional MRI volumes, PET intensities, cerebrospinal fluid (CSF) biomarkers and genetic information as features and implemented random-forest based classification. Additionally, Zhang et al. (2011) conducted a classification based on MRI, PET, and CSF biomarkers. Moreover, other imaging modalities or PET tracers can be considered, as Rondina et al. (2018) used T1-MRI, ^18^F-FDG-PET and rCBF-SPECT as the imaging modalities while Wang et al. (2016) used ^18^F-FDG and ^18^F-florbetapir as tracers of PET.

The studies mentioned above mostly follow three basic steps in the diagnosis algorithms, namely segmentation, feature extraction and classification. During segmentation, data are manually or automatically partitioned into multiple segments based on anatomy or physiology. In this way, the ROIs are well-defined, making it possible to extract features from them. Finally, these features will be fed to the classification step so that the classifiers are able to learn useful diagnostic information and propose predictions for given test subjects. Among them, segmentation plays an important role as it is used to measure the structural metrics in the feature extraction step. However, it is hard to obtain a segmentation automatically and accurately, which leads to a low efficiency. As a result, we proposed an end-to-end diagnosis without segmentation in the following work. What is more, though highly reliable and explainable, these steps could be integrated weakly, as different platforms are used in different steps of these algorithms. The above considerations lead to our attempt to use a neural network in AD diagnosis.

Benefited by the rapid development of computer science and the accumulation of clinical data, deep learning has become a popular and useful method in the field of medical imaging recently. The general applications of deep learning in medical imaging are mainly feature extraction, image classification, object detection, segmentation and registration (Litjens et al., 2017). Among the deep learning networks, convolutional neural networks (CNNs) are common choices. Hosseini-Asl et al. (2016) built a 3D-CNN based on a 3D convolutional auto-encoder, which takes functional MRI (fMRI) images as input and gives the prediction for the AD vs. MCI vs. CN task, while Sarraf and Tofighi (2016) used a CNN structured like LeNet-5 to classify AD from CN based on fMRI. Liu et al. (2018) conducted a T1-MRI and FDG-PET based cascaded CNN, which utilized a 3D CNN to extract features and adopted another 2D CNN to combine multi-modality features for task-specific classification. Previous studies showed a promising potential of AD diagnosis, and thus we propose to use a deep learning framework in our work to complete the feature extraction and classification steps simultaneously.

In this work, we propose a multi-modality AD classifier. It takes both MR and PET images of the hippocampal area as the inputs, and provides predictions in the CN vs. AD task, the CN vs. pMCI task and the sMCI vs. pMCI task. The main contributions of our work are listed below:

(1) We show that segmentation of the key substructures, such as hippocampi, is not a prerequisite in CNN-based classification.
(2) We show that the high-resolution information in the hippocampal area can make up the gap between ROIs of different sizes.
(3) We construct a 3D VGG-variant CNN to implement a single modality AD diagnosis.
(4) We introduce a new framework to combine complementary information from multiple modalities in our proposed network, for the classification tasks of CN vs. AD, CN vs. pMCI and sMCI vs. pMCI.

## Materials and Methods

Studies of biomarkers for AD diagnosis are of great interest in the research fields. Among these bio markers, the shrinkage of the hippocampi is the best-established MRI biomarker to stage the progression of AD (Jack et al., 2011a), and by now the only MRI biomarker qualified for the enrichment of clinical trials (Hill et al., 2014). Therefore, the hippocampi are the most studied organs for MRI based AD diagnosis, and the hippocampal area is chosen to be the ROI of MRI in this work. As for PET images, published studies indicated that AD may cause the decline of ^[18]^F-FDG uptake in both hippocampi and cortices (Mosconi et al., 2006; Mosconi et al., 2008; Jack et al., 2011b). Hence, when dealing with PET images, we tried different ROIs, i.e., containing only hippocampi, and containing both hippocampi and cortices.

### Image Acquisition

Data used in the preparation of this article were obtained from the Alzheimer’s Disease Neuroimaging Initiative (ADNI) database^1^. The ADNI was launched in 2003 as a public-private partnership, led by Principal Investigator Michael W. Weiner, MD. The primary goal of ADNI has been to test whether serial magnetic resonance imaging (MRI), positron emission tomography (PET), other biological markers, and clinical and neuropsychological assessment can be combined to measure the progression of mild cognitive impairment (MCI) and early Alzheimer’s disease (AD). In this work, we used the T1-MRI and the FDG-PET from the baseline and follow-up visit in ADNI, as these two modalities have the greatest number of images. The details about the data acquisition are interpreted on the ADNI website (Jack et al., 2008a). We generated two datasets in this work. The Segmented dataset, containing MR images and corresponding segmentation results, was chosen to verify the effect of the segmentation, and the Paired dataset, containing MR and PET images, to verify the effect of multi-modality images.

In the Segmented dataset, we picked 2861 T1-MR images, including AD and cognitively unimpaired subjects. Basic information of the Segmented dataset is summarized in Table 1. All images in the Segmented dataset were segmented using multi-atlas label propagation with the expectation-maximization (MALP-EM) framework^2^ (Ledig et al., 2015). MALP-EM is a framework for the fully automatic segmentation of MR brain images. The approach is based on multi-atlas label fusion and intensity-based label refinement, using an expectation-maximization (EM) algorithm. Through the MALP-EM framework, we obtained 138 anatomical regions with fixed boundaries, including the hippocampi of interest.

**Table 1:** Summary of the studied subjects from Segmented dataset.

Diagnosis	Number	Age	Gender(M/F)	MMSE
AD	1355	76.13 ± 7.50	772/583	21.89 ± 4.33
CN	1506	76.04 ± 5.81	776/730	29.04 ± 1.20

As for the Paired dataset, we used the following steps to generate it. For the same subject, we paired the MRI with the PET with (a) closest acquisition dates, (b) within 1 year since the MRI scan, and (c) at the time of the scan with the same diagnosis as the MRI. Among the acquired data, the MCI subjects were classified into pMCI and sMCI according to the DSM-5 criteria, that is, MCI should be defined as pMCI if it develops into AD within 3 years, or be defined as sMCI if it does not. Subjects without follow-up data for more than 3 years were ignored. Finally, we acquired 647 AD, 767 MCI (326 pMCI and 441 sMCI) and 731 cognitively unimpaired subjects over 1211 ADNI participants. All the information for these subjects is summarized in Table 2.

**Table 2:** Summary of the studied subjects from the Paired dataset.

Diagnosis	Number	Age	Gender(M/F)	MMSE
AD	647	76.36 ± 7.21	361/287	24.84 ± 2.65
pMCI	326	75.00 ± 7.06	212/114	27.22 ± 1.74
sMCI	441	74.37 ± 7.40	297/144	28.15 ± 1.55
CN	731	76.16 ± 6.02	421/310	28.99 ± 1.20

### Data Processing

The pre-processing of images was implemented by *zxhtools*^3^ (Zhuang et al., 2011). In this work, MR images were re-oriented and resampled to a resolution of 221 × 257 × 221 and with a 1 mm isotropic spacing using *zxhreg* and *zxhtransform* from *zxhtools.* Furthermore, in the Paired dataset, each PET image was rigid-registered to a respective MR image for the proceeding process.

The hippocampal area was selected to be the region of interest (ROI) because of its great significance in AD diagnosis. In addition, due to limited computation ability, we cropped the ROI centered in the hippocampi. For the Segmented dataset, which includes the segmentation results, we directly calculated the center of the hippocampi as it has been shown in the segmentation results. For the Paired dataset, we acquired the central points of the MR images as follows. First, we randomly chose one MR image from the Paired dataset as a template. Then we registered the images from the Segmented dataset to the template image by affine-registration, thus calculating the average indices of the center in the template image. After that, we registered the template image to other MR images in the Paired dataset using affine-transformation and used the corresponding affine matrix to determine the center for each MR image. Finally, each PET image was rigid-registered to a respective MR image for the identification of the hippocampi’s center. After the registration, PET images were transformed into a uniform isotropic spacing of 1 mm.

**FIGURE 1:**
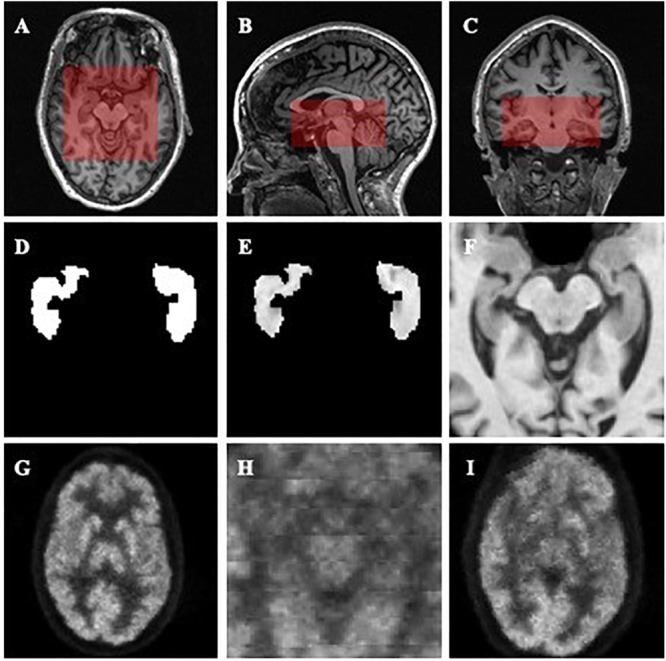
Demonstrations of the datasets and ROIs. **(A–C)** demonstrate the selected ROI of MR images. **(A)** is an axial slice, **(B)** is a sagittal slice, and **(C)** is a coronal slice. **(D**–**F)** are generated from the same MR image to demonstrate the Mask **(D)**, MaskedImage **(E)**, and ImageOnly **(F)** groups. **(D)** is a mask image of the segmentation of hippocampi. **(E)** is a image masked by hippocampal segmentation. **(F)** is a cropped image. **(G–I)** are generated from the same PET image to demonstrate the images in the SmallRF **(H)** and BigRF **(I)** groups, while **(G)** is the corresponding PET image. Among them, **(H)** is cropped from **(G)**, and **(I)** is downsampled from **(G)**.

After the centers of the ROIs were located, we dilated and cropped the ROIs to a region of size 96 × 96 × 48 in voxels from the center of hippocampi for MR images (see the red rectangles in Figure 1A–C). In the experiment on the Segmented dataset, we processed the cropped ROI and corresponding labels in three different ways. Three slightly different groups were obtained: ImageOnly, MaskedImage and Mask. The ImageOnly group contains MR raw images and maintains all the imaging information of the hippocampi and surrounding areas. The MaskedImage group is made up of MR images masked by binary labels, it considers both the original images and the segmentation results for the hippocampi as the inputs. The Mask group is made up of binary hippocampi segmentation labels, only indicating information about the shape and volume of the hippocampi. By comparing the classification performance using these three datasets, it can be judged whether the segmentation results have an important effect on AD diagnosis. The information for the three groups from the Segmented dataset is shown in Figure 1D–F. When it comes to the Paired dataset, we used two different methods to generate the patches of PET images. The group generated using the first method is called the Small Reception Field (SmallRF) group, which has the same reception field as the ROI of MR images with 1 mm isotropic spacing. The group generated using the second method is called the Big Reception Field (BigRF) group, which has the same orientation and ROI center but has a 2 mm isotropic spacing for each dimension, thus having a larger reception field but a lower spatial resolution. The information for the two groups from the Paired dataset is shown in Figure 1H,I as a sample of the original PET image is shown in Figure 1G.

After the data processing, the datasets were randomly split into training sets, validation sets, and testing sets according to the patient IDs to ensure that all subjects of the same patient only appear in one set. Finally, 70% of a dataset was used as the training set, 10% as the validation set, and 20% as the testing set by random sampling. Details of these subsets were shown in Supplementary Tables S1 and S2.

### Methodology

Convolutional neural network (LeCun et al., 1995) is a deep feedforward neural network composed of multi-layer artificial neurons, with excellent performance in large-scale image processing. Unlike traditional methods which use manually extracted features of radiological images, CNNs are used to learn general features automatically. CNNs are trained with a back propagation algorithm while it usually consists of multiple convolutional layers, pooling layers and fully connected layers and connects to the output units through fully connected layers or other kinds of layers. Compared to other deep feedforward networks, CNNs have fewer connections and a smaller number of parameters, due to the sharing of the convolution kernel among pixels and are therefore easier to train and more popular.

With CNNs prospering in the field of computer vision, a number of attempts have been made to improve the original network structure to achieve better accuracy. VGG (Simonyan and Zisserman, 2014) is a neural network based on AlexNet (Krizhevsky et al., 2012) and it achieved a 7.3% error rate in the 2014 ILSVRC competition (Russakovsky et al., 2015) as one of the Top-5 winners. VGGs further deepen the network based on AlexNet by adding more convolutional layers and pooling layers. Different from traditional CNNs, VGGs evaluate very deep convolutional networks for large-scale image classification, which come up with significantly more accurate CNN architectures and can achieve excellent performance even when used as a part of relatively simple pipelines. In this work, we built our network with reference to the structure of VGG.

## Experiments

In the Section “Data Type Analysis”, we determined the proper types of data and ROIs through two experiments. In the Section “Multi-Modality AD Classifier”, we constructed a set of VGG-like multi-modality AD classifiers, which considers both T1-MRI and FDG-PET data as inputs and provides predictions. In the Section “Classification of sMCI vs. pMCI and CN vs. pMCI Tasks”, we trained and tested our networks with the pMCI and sMCI data. Finally, in the Section “Comparison With Other Methods” we compared our proposed method with state-of-the-art methods.

### Implementation Details

All the networks mentioned above were programmed based on TensorFlow (Abadi et al., 2016). Training procedures of the networks were conducted on a personal computer with a Nvidia GTX1080Ti GPU. During the training, batch normalization (Ioffe and Szegedy, 2015) was deployed in the convolutional layers and dropout (Hinton et al., 2015) was deployed in fully connected layers to avoid overfitting. To accelerate the training process and to avoid local minima, we used an ADAM optimizer (Kingma and Ba, 2014) to train. The batch size was set to 16 when we trained single modality networks and to eight when we trained multi-modality networks. The number of epochs was set to 150, though the loss would generally converge after 30 epochs. Each training epoch took several minutes. During training, the parameters of the networks were saved every 10 epochs. The resulting models were tested using the validation data set. The accuracies and receiver operating characteristic (ROC) curves of the classification on the validation data were then calculated, and the model with the best accuracy was chosen to be the final classifier.

### Data Type Analysis

In order to determine the proper data type for network training, we designed two experiments and evaluated the classification performances of models when they were fed with different data types.

(1) Testing whether segmentation is needed in the MR images. We used three different groups from the Segmented Dataset, with or without segmentation, to show that segmentation is not necessary for a CNN.
(2) Finding a proper PET ROI. Different spacings for PET images, i.e., the SmallRF and the BigRF groups from the Paired Dataset, were tested and we found that the classification model with the SmallRF group is similar to the model with the BigRF group in performance.

**FIGURE 2:**
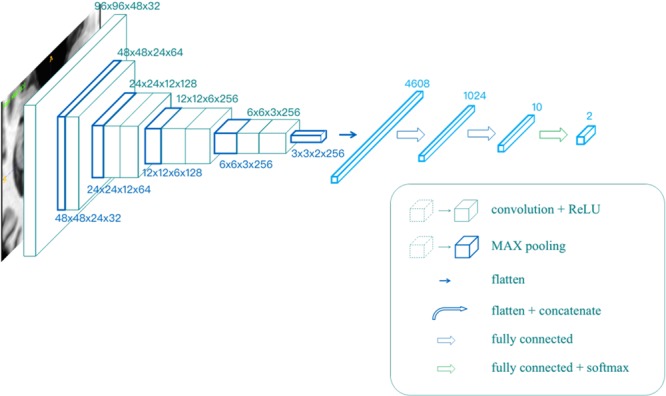
The architecture of the single modality classifier.

All the models mentioned above were trained in the same network, as shown in Figure 2. The input resolution is 96 × 96 × 48 in voxels, and the network contains eight convolutional layers, five max-pooling layers, and three fully connected layers. The output was given through a softmax layer.

#### The Influence of Segmentation

As mentioned above, segmentation plays an important role in traditional classification methods. However, segmentation is also known to be time-consuming. Additionally, CNN can extract useful features directly from raw images, as CNNs show a strong ability to locate key points in object detection tasks for natural images (Ren et al., 2015; He et al., 2017).

To verify the effect of segmentation, we segmented the AD and cognitively unimpaired subjects of T1-MR images with the MALP-EM algorithm (Ledig et al., 2015) and obtained the Segmented datasets, including 2861 subjects and containing both MR images and the corresponding segmentation. In our assumption, segmentation can indicate the shapes, volumes, textures and relative locations of hippocampal areas. Therefore, the data obtained from the subjects formed three different groups, as shown in Figure 1D–F. The ImageOnly group contains raw MR images only; the Mask group is made up of binary hippocampal segmentation labels and the MaskedImage group is made up of MR images masked by the binary labels.

For each model trained from these groups, accuracy and AUC were evaluated, as listed in Table 3. Among all the three models, the model trained by the Mask group provided a favorable prediction, though inferior to those trained by the ImageOnly and the MaskedImage group. The results indicate that segmentation results do contain information needed for the classification, however, it is not necessary for the classification task since CNN is able to learn useful features without labeling the voxels. In addition, features from the region out of the hippocampi also provide further information to separate AD patients from normal ones.

**Table 3:** Summary of the models trained from the Mask, MaskedImage, and ImageOnly groups for CN vs. AD task.

MRI ROI	ACC^1^	SEN	SPE	AUC
Mask	76.57%	83.87%	71.51%	84.24%
Maskedlmage	79.21%	76.61%	81.01%	84.63%
ImageOnly	84.82%	87.90%	82.68%	87.47%

*The Segmented dataset was used. ^*1*^ACC, SEN, SPE, AUC denotes accuracy, sensitivity, specificity and area under curve, respectively. When testing, the numbers of true positive (TP), true negative (TN), false negative (FP), and false negative (FN) subjects were counted, as ACC = (TP+TN)/(TP+TN+FP+FN), SEN = TP/(TP+FN), SPE = TN/(TN+FP). AUC is obtained through calculating the area under the receiver operating characteristic (ROC) curve. For all four metrics, the values are between 0 and 100%, the higher, the better.*

#### ROI Determination for PET Images

Due to the limitation of GPU RAM and its computational ability, it was difficult to consider the entire image as the network input, as our proposed network only considered a region of 96 × 96 × 48 in voxels, which was still 2.91 times the input size of the original VGG (224 × 224 pixels × 3 channels). Hence, the selection of the ROI was of great importance, as only the features in the ROI were considered. As for the MR images, the selection of the ROI was of little doubt, because the hippocampal area was long enough to be the main concern of AD research (Jack et al., 2011b; Hill et al., 2014). However, the ROIs of PET images varied, as studies also attached great significance to metabolic changes in cortices, e.g., temporal lobes (Mosconi et al., 2006, 2008).

To verify the effects of cortices on the classification, we generated two groups from all PET images from the Paired dataset, the SmallRF and the BigRF groups, as shown in Figure 1H,I. The SmallRF group uses exactly the same reception field with the MRI ROI; the images in the BigRF group are eight times the volume of the images in the SmallRF group but have a lower spatial resolution.

Two models were trained using these two groups, and their performance was evaluated by some metrics, as listed in Table 4. The result showed that the two models behaved similarly. This is because although the SmallRF group has a higher spatial resolution, the BigRF group contains more features. Furthermore, in terms of multi-modality classification tasks, the SmallRF group might be better, because PET images in the SmallRF group were voxel-wisely aligned with paired MR images, which could help better locate the spatial features. Therefore, we chose the same ROI for both MR and PET images in the following experiments (see the red rectangles in Figure 1A–C).

**Table 4:** Summary of the models trained from the SmallRF and the BigRF groups for CN vs. AD task.

PET ROI	ACC	SEN	SPE	AUC
SmallRF PET	89.11%	90.24%	87.77%	92.69%
BigRF PET	89.44%	87.20%	92.09%	90.35%

*The Paired dataset was used.*

### Multi-Modality AD Classifier

The information a classifier can obtain, by using a single modality, is limited, as one medical imaging method can only profile one or several aspects of AD pathological changes, which is far from being complete. For example, T1-MR images provide a high-resolution brain structure but give little information about the functional information of the brain. Meanwhile, FDG-PET images are fuzzy but are better in revealing the metabolic activity of glucose in the brain. In order to take as much information of the brain as possible, we introduced a classification framework to integrate multi-modality information.

To prepare the dataset, we first matched MR with PET images and transformed them into same world coordinates. After that, paired images of MR and PET were aligned by rigid registration to ensure that the voxels of the same indices in the paired images represent the same part of the brain. After the paired images were cropped with reference to the center point of MR images, the Paired dataset was obtained.

**FIGURE 3:**
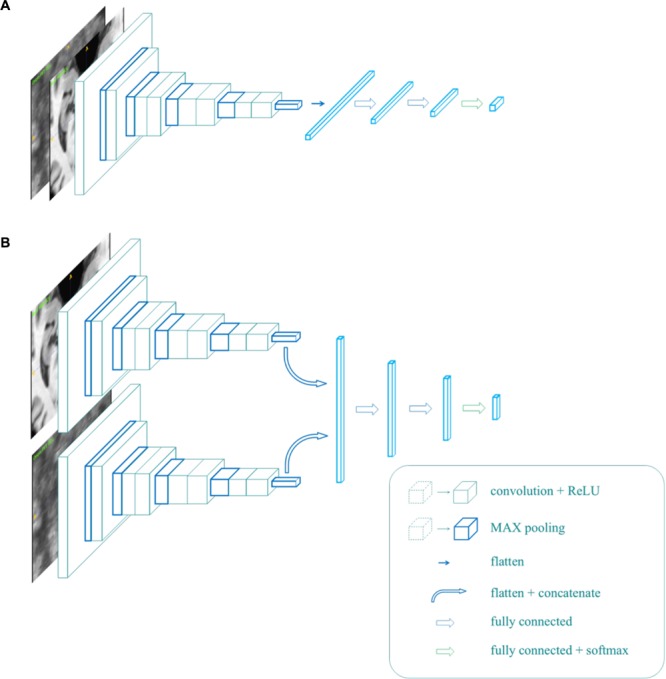
The architecture of the multi-modality network **(A,B)**.

To implement the multi-modality classifier, we proposed two different network architectures, as shown in Figure 3. In Figure 3A, MR and PET images were used as two parallel channels, in which paired images were stacked into 4D images. In these 4D images, the first three dimensions represent the three spatial dimensions, and the fourth one represents the channels. In Figure 3B, MR and PET images have separate entrances, as they are convolved, respectively, in two separate VGG-11s, and the extracted features are concatenated. This network was trained in two strategies, denoted by B1 and B2. B1 was to train the model with weights shared for the convolutional layers. Meanwhile, B2 usedwas to update the weights of two VGG-11s separately.

**FIGURE 4:**
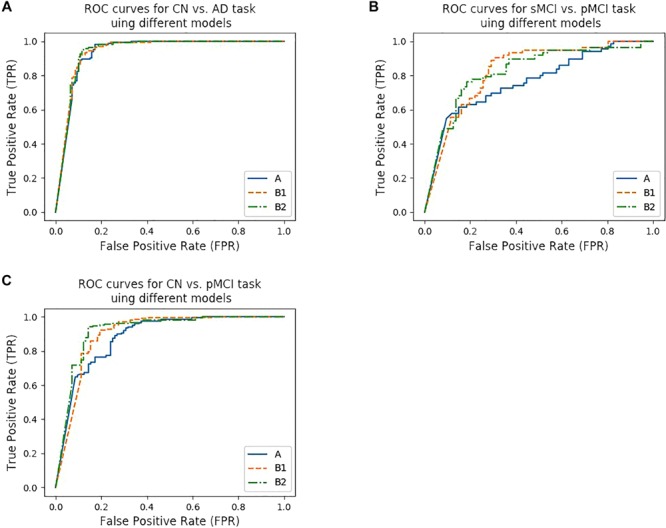
ROC curves of different models. **(A**–**C)** show the ROC curves for three tasks using different models. **(A)** shows the ROC curves for CN vs. AD task using model trained from protocol A, B1, and B2, while **(B)** shows the ROC curves for CN vs. pMCI task, **(C)** shows the ROC curves for sMCI vs. pMCI task, respectively.

We trained five models based on the Paired dataset, that is, two single modality models (for MRI and PET respectively), and three multi-modality models (A, B1, and B2). The results are shown in Table 5 and Figure 4A. As shown in Table 5, multi-modality classifiers had better performance than single modality classifiers. Additionally, among the three multi-modality models, the model trained with strategy B1 had the highest accuracy and sensitivity, while the model trained with strategy B2 had the highest specificity and AUC.

**Table 5:** Summary of the models trained from single modality protocols and three multi-modality protocols for CN vs. AD task.

Method	ACC	SEN	SPE	AUC
MRI	81.19%	79.27%	83.45%	83.67%
PET	89.11%	90.24%	87.77%	**92.69%**
A	87.79%	85.98%	**89.93%**	89.42%
B1	**90.10%**	**90.85%**	89.21%	90.84%
B2	89.44%	89.02%	**89.93%**	92.01%

*The Paired dataset was used. The best results were indicated in bold.*

### Classification of sMCI vs. pMCI and CN vs. pMCI Tasks

Simply classifying AD patients from normal controls is relatively easy but of little significance, as the development of AD can be observed easily by the behaviors of the patients. In addition, there are a lot of alternative indicators in clinical diagnosis. Therefore, the prediction of AD seems to be more meaningful, as one of the main concerns is telling pMCI from sMCI and normal individuals. As pMCI would progress to AD while the other two would not, identifying pMCI could give a prediction of the development of MCI, and thus have high reference value and clinical meaning.

According to Lin et al. (2018), the models that were trained by the CN vs. AD training set performed better than the models trained by the sMCI vs. pMCI training set in the sMCI vs. pMCI task. Therefore, we trained models with the CN vs. AD training set and tested the models with the CN vs. pMCI testing set and the sMCI vs. pMCI testing set, with the results shown in Table 6 and Figure 4B,C. Though B1 performed slightly better in CN vs. AD task, B2 was superior in CN vs. pMCI and sMCI vs. pMCI tasks. These results indicate that features of MRI and PET tend to be more consistent when dementia is highly developed, since convolutional kernels of model B1 shared the weight, while those of B2 did not.

**Table 6:** Summary of the models trained from three multi-modality protocols for CN vs. AD.

Method	A				B1				B2			
	ACC	SEN	SPE	AUC	ACC	SEN	SPE	AUC	ACC	SEN	SPE	AUC
CN/AD	87.79%	85.98%	**89.93%**	89.42%	**90.10%**	**90.85%**	89.21%	90.84%	89.44%	89.02%	**89.93%**	**92.01%**
CN/pMCI	70.49%	73.17%	65.00%	71.63%	79.10%	**87.80%**	61.25%	76.84%	**82.38%**	87.20%	**72.50%**	**81.64%**
sMCI/pMCI	65.28%	65.63%	65.00%	65.81%	65.28%	54.69%	**73.75%**	66.82%	**72.22%**	**73.44%**	71.25%	**77.49%**

*The best results were indicated in bold.*

### Comparison With Other Methods

In this part, we compared our method with those that were used in previous literature. We first compared our method with state-of-the-art research using 3D CNN-based multi-modality models as well (Lin et al., 2018). Liu et al. (2015) proposed a multi-modality cascaded CNN. They used the patch-based information of a whole brain to train or test their models and they integrated the information from the two modalities by concatenating the feature maps(Liu et al., 2015). Table 7 shows the results of the method in comparison to our work. Note that our models used the data from multiple facilities and that our models only used the hippocampal area as the input. These would influence the behavior of our method.

**Table 7:** Comparison of our proposed method and Liu’s multi-modality method.

Method	Subjects	Modality	CN vs. AD				CN vs. pMCI			
			ACC	SEN	SPE	AUC	ACC	SEN	SPE	AUC
Liu et al., 2018	93 AD + 204 MCI + 100 CN	MRI	84.97%	82.65%	87.37%	90.63%	77.84%	76.81%	78.59%	82.72%
		PET	88.08%	90.70%	85.98%	94.51%	78.41%	77.94%	78.70%	85.96%
		Both	**93.26%**	**92.55%**	**93.94%**	**95.68%**	82.95%	81.08%	**84.31%**	**88.43%**
Proposed method	465 AD + 567 MCI + 480 CN	MRI	81.19%	79.27%	83.45%	83.67%	–	–	–	–
		PET	89.11%	90.24%	87.77%	92.69%	–	–	–	–
		Both	90.10%^1^	90.85%	89.21%	90.84%	82.38%^2^	87.20%	72.50%	81.64%
		Both	–	–	–	–	**87.46%**^3^	**90.73%**	80.61%	87.61%

*^*1*^Using B1 protocol, the CN vs. AD training set and the CN vs. AD testing set. ^*2*^Using B2 protocol, the CN vs. pMCI training set and the CN vs. pMCI testing set. ^*3*^Using B1 protocol, the CN vs. AD training but the CN vs. pMCI testing set. See Table 9 for reference. The best results were indicated in bold.*

Moreover, Lin et al. (2018), chose to reduce the amount of input by slicing the data (in different directions) instead of cropping the hippocampi out as we did. Tong et al. (2017) used non-linear graph fusion to join the features of different modalities. In Zu et al.’s (2016) study, the feature selection from multiple modalities were treated as different learning tasks. Liu et al. (2015) used stacked autoencoders (SAE) with a masking training strategy. Jie et al. (2015) used a manifold regularized multitask feature learning method to preserve both the relations among modalities of data and the distribution in each modality. Li et al. (2014) used a deep learning framework to predict the missing data. Table 8 compares the previous multi-modality models with our proposed models. Among all the results listed below, our results are favorable in the CN vs. AD task and are the best in the sMCI vs. pMCI task.

**Table 8:** Comparison of our proposed method and published AD diagnosis methods.

Method	Subjects	CN vs. AD				sMCI vs. pMCI			
		ACC	SEN	SPE	AUC	ACC	SEN	SPE	AUC
Lin et al., 2018	93 AD + 204 MCI + 100 CN	88.79%	–	–	–	73.04%	–	–	–
Tong et al., 2017	37 AD + 75 MCI + 35 CN	88.6%	–	–	94.8%	–	–	–	–
Zu et al., 2016	51 AD + 99MCI + 52 CN	**95.95%**	–	–	–	69.78%	–	–	–
Liu et al., 2015	85 AD + 168 MCI + 77 CN	91.40%	92.32%	90.42%	–	–	–	–	–
Jie et al., 2015	51 AD + 99 MCI + 52 CN	95.03%	–	–	–	68.94%	–	–	–
Li et al., 2014	93 AD + 204 MCI + 101 CN	92.87%	–	–	89.82%	72.44%	–	–	70.14%
Proposed method	465 AD + 567 MCI + 480 CN	90.10%^1^	90.85%	89.21%	90.84%	72.22%^2^	**73.44%**	71.25%	77.49%
		–	–	–	–	**76.90%**^3^	68.15%	**83.93%**	**79.61%**

*^*1*^Using B1 protocol, the CN vs. AD training set and the CN vs. AD testing set. ^*2*^Using B2 protocol, the CN vs. sMCI training set and the CN vs. sMCI testing set. ^*3*^Using B2 protocol, the CN vs. AD training but the CN vs. sMCI testing set. See Table 9 for reference. The best results were indicated in bold.*

## Discussion

In this work, we proposed a VGG-like framework, with several instances, to implement a T1-MRI and FDG-PET based multi-modality AD diagnosing system. The ROI of MRI was selected to be the hippocampal area, as it is the most frequently studied and is thought to be of the highest clinical value. Through the experiments, we proved that segmentation is not necessary for a CNN-based diagnosing system, which is different from the traditional machine learning based methods. However, registration is still needed, as the images we used were taken from different facilities and had different spacings and orientations. Although models obtained from the SmallRF and BigRF groups had similar performances, the ROI of PET was chosen to be the same as the MRI’s, because the ROI of SmallRF was voxel-wisely aligned with the ROI of the paired MRI. In short, only hippocampal areas were used as ROIs in our proposed methods, which is the main difference between our study and previous studies. Thus, we constructed a deeper neural network and fed it with medical images of higher resolution, as we supposed that the hippocampal area itself can serve as a favorable reference in AD diagnosis.

”Since the ROI was selected, we introduced a multi-modality method to the classifier. Two networks and three types of models were proposed as listed in Table 6. Among these three types of models, the model trained using strategy B1, which means that the MR and PET images were separately input for the convolutional layers, but with their convolutional kernels shared, performed the best in the CN vs. AD task. One possible explanation is that MR and PET images have some common features, and sharing weight helped the model to extract these features during the training process. Furthermore, we used proposed networks to train CN vs. pMCI and sMCI vs. pMCI classifiers, both of them indicated the potential of preclinical diagnosis using our proposed methods.

We also followed Lin et al.’s (2018) lead and used the model trained by CN vs. AD subjects to distinguish sMCI and pMCI. The results were better than that of the model trained by sMCI and pMCI themselves, as shown in Table 9. This is reasonable because the features of sMCI and pMCI are close to each other in the feature space and are difficult to differentiate, while those of CN and AD are widely spread making the classification a lot easier. The same conclusion can be obtained by testing the CN vs. AD model on the CN vs. pMCI dataset. Specifically, when the CN vs. AD model was used, the accuracy reached 76.90% for sMCI vs. pMCI and 87.46% for CN vs. pMCI, which was about 5% higher than the accuracy obtained using their own models. These results are also better than those of Lin et al.’s (2018).

**Table 9:** Comparison of the performance of models trained from the CN vs. AD training set and the tasks’ own training set.

Task	Training Set	Testing Set	B1				B2			
			ACC	SEN	SPE	AUC	ACC	SEN	SPE	AUC
CN/pMCI	CN/AD	CN/pMCI	87.46%	90.73%	80.61%	87.61%	87.13%	87.80%	85.71%	90.31%
CN/pMCI	CN/pMCI	CN/pMCI	79.10%	87.80%	61.25%	76.84%	82.38%	87.20%	72.50%	81.64%
sMCI/pMCI	CN/AD	sMCI/pMCI	73.60%	66.67%	79.17%	75.59%	76.90%	68.15%	83.93%	79.61%
sMCI/pMCI	sMCI/pMCI	sMCI/pMCI	65.28%	54.69%	73.75%	66.82%	72.22%	73.44%	71.25%	77.49%

*The Paired dataset was used.*

As for the future work, we only used two modalities (T1-MRI and FDG-PET) as inputs for this work. However, new modalities can easily be implemented based on the proposed networks. The interested new imaging modalities include T2-MRI (Rombouts et al., 2005), ^11^C-PIB-PET (Zhang et al., 2014), and other PET agents such as amyloid protein imaging (Glenner and Wong, 1984). Also, the features extracted by CNN are hard for human beings to comprehend, while some methods like attention mechanisms (Jetley et al., 2018) are able to visualize and analyze the activation maps of the model, in which future work could be done to improve the classification performance and to discover new medical imaging biomarkers.

## Conclusion

To conclude, we have proposed a multi-modality CNN-based classifier for AD diagnosis and prognosis. VGG backbone, which is deeper than most similar studies, has been used and explored. The accuracy of models reached 90.10% for the CN vs. AD task, 87.46% for the CN vs. pMCI task and 76.90% for the sMCI vs. pMCI task. Our work also indicates that the hippocampal area with no segmentation can be chosen as the input.

## Ethics Statement

Data used in the preparation of this article were obtained from the Alzheimer’s Disease Neuroimaging Initiative (ADNI) database (adni.loni.usc.edu). The ADNI was launched in 2003 as a public-private partnership, led by Principal Investigator Michael W. Weiner, MD. The primary goal of ADNI has been to test whether serial magnetic resonance imaging (MRI), positron emission tomography (PET), other biological markers, and clinical and neuropsychological assessment can be combined to measure the progression of mild cognitive impairment (MCI) and early Alzheimer’s disease (AD). Since its launch more than a decade ago, the landmark public-private partnership has made major contributions to AD research, enabling the sharing of data between researchers around the world. For up-to-date information, see www.adni-info.org.

## Author Contributions

XZ is the corresponding author. XZ proposed the idea, supervised and managed the research, and revised the manuscript. YH lead the implementations, experiments, and wrote the manuscript. JX co-lead the work and wrote the manuscript. YZ provided the support to the work of coding, experiments, and wrote the manuscript. TT co-investigated the work and revised the manuscript.

## Conflict of Interest Statement

The authors declare that the research was conducted in the absence of any commercial or financial relationships that could be construed as a potential conflict of interest.

## Footnotes

1 http://adni.loni.usc.edu

2 https://biomedia.doc.ic.ac.uk/software/malp-em/

3 http://www.sdspeople.fudan.edu.cn/zhuangxiahai/0/zxhproj/

## References

[B1] Abadi M. Barham P. Chen J. Chen Z. Davis A. Dean J. (2016). “Tensorflow: a system for large-scale machine learning,” in *Proceedings of the 12th USENIX Conference on Operating Systems Design and Implementation OSDI’16* Savannah, GA 265–283.

[B2] American Psychiatric Association (2013). *Diagnostic and Statistical Manual of Mental Disorders (DSM-5)*. Philadelphia: American Psychiatric Pub.

[B3] Ardekani B. A. Bermudez E. Mubeen A. M. Bachman A. H. Alzheimer’s Disease Neuroimaging Initiative. (2017). Prediction of incipient Alzheimer’s disease dementia in patients with mild cognitive impairment. *J. Alzheimer’s Dis.* 55 269–281. 27662309 10.3233/JAD-160594

[B4] Cabral C. Silveira M. (2013). “Classification of Alzheimer’s disease from FDG-PET images using favourite class ensembles,” in *Proceedings of the 35th Annual International Conference of the IEEE Engineering in Medicine and Biology Society (EMBC)* (Osaka: IEEE) 2477–2480. 10.1109/EMBC.2013.6610042 24110229

[B5] Cárdenas-Peña D. Collazos-Huertas D. Castellanos-Dominguez G. (2017). Enhanced data representation by kernel metric learning for dementia diagnosis. *Front. Neurosci.* 11:413 10.3389/fnins.2017.00413 PMC5526982 28798659

[B6] Correa N. M. Li Y. O. Adali T. Calhoun V. D. (2009). “Fusion of fMRI, sMRI, and EEG data using canonical correlation analysis,” in *Proceedings of the 2009 IEEE International Conference on Acoustics, Speech and Signal Processing* (Taipei: IEEE) 385–388.

[B7] Desikan R. S. Cabral H. J. Hess C. P. Dillon W. P. Glastonbury C. M. Weiner M. W. (2009). Automated MRI measures identify individuals with mild cognitive impairment and Alzheimer’s disease. *Brain* 132 2048–2057. 10.1093/brain/awp123 19460794 PMC2714061

[B8] Glenner G. Wong C. W. (1984). Alzheimer’s disease: initial report of the purification and characterization of a novel cerebrovascular amyloid protein. *Biochem. Biophys. Res. Commun.* 120 885–890. 10.1016/s0006-291x(84)80190-4 6375662

[B9] Gray K. R. Aljabar P. Heckemann R. A. Hammers A. Rueckert D. (2013). Random forest-based similarity measures for multi-modal classification of Alzheimer’s disease. *NeuroImage* 65 167–175. 10.1016/j.neuroimage.2012.09.065 23041336 PMC3516432

[B10] Harada R. Okamura N. Furumoto S. Tago T. Maruyama M. Higuchi M. (2013). Comparison of the binding characteristics of [18 F] THK-523 and other amyloid imaging tracers to Alzheimer’s disease pathology. *Eur. J. Nucl. Med. Mol. Imaging* 40 125–132. 10.1007/s00259-012-2261-2 23100049

[B11] He K. Gkioxari G. Dollár P. Girshick R. (2017). Mask r-cnn. *arXiv* 10.1109/TPAMI.2018.2844175 29994331

[B12] Hill D. L. G. Schwarz A. J. Isaac M. Pani L. Vamvakas S. Hemmings R. (2014). Coalition against major diseases/european medicines agency biomarker qualification of hippocampal volume for enrichment of clinical trials in predementia stages of Alzheimer’s disease. *Alzheimer’s Dement.* 10 421–429. 10.1016/j.jalz.2013.07.003 24985687

[B13] Hinton G. E. Srivastava N. Krizhevsky A. Sutskever I. Salakhutdinov R. R. (2015). Improving neural networks by preventing co-adaptation of feature detectors. *arXiv*

[B14] Hosseini-Asl E. Gimel’farb G. El-Baz A. (2016). Alzheimer’s disease diagnostics by a deeply supervised adaptable 3d convolutional network. *arXiv* 10.2741/4606 28930562

[B15] Huang C. Wahlund L. O. Almkvist O. Elehu D. Svensson L. Jonsson T. (2003). Voxel-and VOI-based analysis of SPECT CBF in relation to clinical and psychological heterogeneity of mild cognitive impairment. *Neuroimage* 19 1137–1144. 10.1016/s1053-8119(03)00168-x 12880839

[B16] Ioffe S. Szegedy C. (2015). Batch normalization: accelerating deep network training by reducing internal covariate shift. *arXiv*

[B17] Jack C. R. Jr. Albert M. S. Knopman D. S. McKhann G. M. Sperling R. A. Carrillo M. C. (2011a). Introduction to the recommendations from the national institute on aging-alzheimer’s association workgroups on diagnostic guidelines for alzheimer’s disease. *Alzheimer’s Dement.* 7 257–262. 10.1016/j.jalz.2011.03.004 21514247 PMC3096735

[B18] Jack C. R. Jr. Barkhof F. Bernstein M. A. Cantillon M. Cole P. E. DeCarli C. (2011b). Steps to standardization and validation of hippocampal volumetry as a biomarker in clinical trials and diagnostic criterion for Alzheimer’s disease. *Alzheimer’s Dement.* 7 474–485. 21784356 10.1016/j.jalz.2011.04.007 PMC3396131

[B19] Jack C. R. Jr. Bernstein M. A. Fox N. C. Thompson P. Alexander G. Harvey D. (2008a). The Alzheimer’s disease neuroimaging initiative (ADNI): MRI methods. *J. Mag. Reson. Imag.* 27 685–691. 10.1002/jmri.21049 PMC2544629 18302232

[B20] Jack C. R. Jr. Lowe V. J. Senjem M. L. Weigand S. D. Kemp B. J. Shiung M. M. (2008b). 11C PiB and structural MRI provide complementary information in imaging of Alzheimer’s disease and amnestic mild cognitive impairment. *Brain* 131 665–680. 10.1093/brain/awm336 18263627 PMC2730157

[B21] Jetley S. Lord N. A. Lee N. Torr P. H. S. (2018). Learn to pay attention. *arXiv*

[B22] Jie B. Zhang D. Cheng B. Shen D. (2015). Manifold regularized multitask feature learning for multimodality disease classification. *Hum. Brain Mapp.* 36 489–507. 10.1002/hbm.22642 25277605 PMC4470367

[B23] Kingma D. P. Ba J. (2014). Adam: a method for stochastic optimization. *arXiv* 10.1002/mp.13112

[B24] Krizhevsky A. Sutskever I. Hinton G. E. (2012). Imagenet classification with deep convolutional neural networks. *Adv. Neural Inform. Process. Syst.* 25 1097–1105.

[B25] Lebedeva A. K. Westman E. Borza T. Beyer M. K. Engedal K. Aarsland D. (2017). MRI-based classification models in prediction of mild cognitive impairment and dementia in late-life depression. *Front. Aging Neurosci.* 9:13 10.3389/fnagi.2017.00013 PMC5288688 28210220

[B26] LeCun Y. Jackel L. D. Bottou L. Cortes C. Denker J. S. Drucker H. (1995). “Learning algorithms for classification: A comparison on handwritten digit recognition,” in *Neural Networks: The Statistical Mechanics Perspective* eds Oh J. H. Kwon C. Cho S. (Singapore: World Scientific) 261–276.

[B27] Ledig C. Heckemann R. A. Hammers A. Lopez J. C. Newcombe V. F. J. Makropoulos A. (2015). Robust whole-brain segmentation: application to traumatic brain injury. *Med. Image Anal.* 21 40–58. 10.1016/j.media.2014.12.003 25596765

[B28] Li R. Zhang W. Suk H. I. Wang L. Li J. Shen D. (2014). “Deep learning based imaging data completion for improved brain disease diagnosis,” in *Proceedings of the International Conference on Medical Image Computing and Computer-Assisted Intervention* (Cham: Springer) 305–312. 10.1007/978-3-319-10443-0_39 PMC4464771 25320813

[B29] Lin W. Tong T. Gao Q. Guo D. Du X. Yang Y. (2018). Convolutional neural networks-based MRI image analysis for the alzheimer’s disease prediction from mild cognitive impairment. *Front. Neurosci.* 12:777 10.3389/fnins.2018.00777 PMC6231297 30455622

[B30] Litjens G. Kooi T. Bejnordi B. E. Adiyoso Setio A. A. Ciompi F. Ghafoorian M. (2017). A survey on deep learning in medical image analysis. *Med. Image Anal.* 42 60–88. 10.1016/j.media.2017.07.005 28778026

[B31] Liu M. Cheng D. Wang K. Wang Y. (2018). Multi-modality cascaded convolutional neural networks for alzheimer’s disease diagnosis. *Neuroinformatics* 16 295–308. 10.1007/s12021-018-9370-4 29572601

[B32] Liu S. Liu S. Cai W. Che H. Pujol S. Kikinis R. (2015). Multimodal neuroimaging feature learning for multiclass diagnosis of Alzheimer’s disease. *IEEE Trans. Biomed. Eng.* 62 1132–1140. 10.1109/tbme.2014.2372011 25423647 PMC4394860

[B33] Mosconi L. De Santi S. Li Y. Zhan J. Tsui W. H. Boppana M. (2006). Visual rating of medial temporal lobe metabolism in mild cognitive impairment and Alzheimer’s disease using FDG-PET. *Eur. J. Nucl. Med. Mol. Imaging* 33 210–221. 10.1007/s00259-005-1956-z 16311757

[B34] Mosconi L. Tsui W. H. Herholz K. Pupi A. Drzezga A. Lucignani G. (2008). Multicenter standardized 18F-FDG PET diagnosis of mild cognitive impairment, Alzheimer’s disease, and other dementias. *J. Nuclear Med.* 49:390 10.2967/jnumed.107.045385 PMC3703818 18287270

[B35] Patterson C. (2018). *World Alzheimer Report 2018 The State of the Art of Dementia Research: New frontiers*. London: Alzheimer’s Disease International (ADI).

[B36] Petersen R. C. Lopez O. Armstrong M. J. Getchius T. S. D. Ganguli M. Gloss D. (2018). Practice guideline update summary: mild cognitive impairment: report of the guideline development, dissemination, and implementation subcommittee of the American academy of neurology. *Neurology* 90 126–135. 10.1212/wnl.0000000000004826 29282327 PMC5772157

[B37] Petersen R. C. Smith G. E. Waring S. C. (1999). Mild cognitive impairment: clinical characterization and outcome. *Arch. Neurol.* 56 303–308. 10190820 10.1001/archneur.56.3.303

[B38] Ren S. He K. Girshick R. Sun J. (2015). “Faster r-cnn: Towards real-time object detection with region proposal networks,” in *Proceedings of the IEEE Transactions on Pattern Analysis and Machine Intelligence* (Piscataway: IEEE). 10.1109/TPAMI.2016.2577031 27295650

[B39] Rombouts S. A. R. B. Barkhof F. Goekoop R. Stam C. J. Scheltens P. (2005). Altered resting state networks in mild cognitive impairment and mild Alzheimer’s disease: an fMRI study. *Hum. Brain Mapp.* 26 231–239. 10.1002/hbm.20160 15954139 PMC6871685

[B40] Rondina J. M. Ferreira L. K. de Souza Duran F. L. Kubo R. Ono C. R. Leite C. C. (2018). Selecting the most relevant brain regions to discriminate Alzheimer’s disease patients from healthy controls using multiple kernel learning: a comparison across functional and structural imaging modalities and atlases. *NeuroImage Clin.* 17 628–641. 10.1016/j.nicl.2017.10.026 29234599 PMC5716956

[B41] Russakovsky O. Deng J. Su H. Krause J. Satheesh S. Ma S. (2015). Imagenet large scale visual recognition challenge. *Int. J. Comp. Vis.* 115 211–252. 10.1007/s11263-015-0816-y

[B42] Samper-González J. Burgos N. Bottani S. Fontanella S. Lu P. Marcoux A. (2018). Reproducible evaluation of classification methods in Alzheimer’s disease: framework and application to MRI and PET data. *bioRxiv*10.1016/j.neuroimage.2018.08.042 30130647

[B43] Sarraf S. Tofighi G. (2016). Classification of alzheimer’s disease using fmri data and deep learning convolutional neural networks. *arXiv*

[B44] Silveira M. Marques J. (2010). “Boosting Alzheimer disease diagnosis using PET images,” in *Proceedings of the 2010 20th International Conference on Pattern Recognition* (Istanbul: IEEE) 2556–2559.

[B45] Simonyan K. Zisserman A. (2014). Very deep convolutional networks for large-scale image recognition. *arXiv*

[B46] Sorensen L. Igel C. Pai A. Balas I. Anker C. Lillholm M. (2017). Differential diagnosis of mild cognitive impairment and Alzheimer’s disease using structural MRI cortical thickness, hippocampal shape, hippocampal texture, and volumetry. *NeuroImage Clin.* 13 470–482. 10.1016/j.nicl.2016.11.025 28119818 PMC5237821

[B47] Tong T. Gray K. Gao Q. Chen L. Rueckert D. (2017). Multi-modal classification of Alzheimer’s disease using nonlinear graph fusion. *Patt. Recogn.* 63 171–181. 10.1016/j.patcog.2016.10.009

[B48] Wang P. Chen K. Yao L. Hu B. Wu X. Zhang J. (2016). Multimodal classification of mild cognitive impairment based on partial least squares. *J. Alzheimer’s Dis.* 54 359–371. 10.3233/jad-160102 27567818

[B49] World Health Organization (2012). *Dementia: a Public Health Priority*. Geneva: World Health Organization.

[B50] Zhang D. Wang Y. Zhou L. Yuan H. Shen D. (2011). Multimodal classification of Alzheimer’s disease and mild cognitive impairment. *Neuroimage* 55 856–867. 10.1016/j.neuroimage.2011.01.008 21236349 PMC3057360

[B51] Zhang S. Smailagic N. Hyde C. Noel-Storr A. H. Takwoingi Y. McShane R. (2014). 11 C-PIB-PET for the early diagnosis of Alzheimer’s disease dementia and other dementias in people with mild cognitive impairment (MCI). *Coch. Database Syst. Rev.* 2014:CD010386. 10.1002/14651858.CD010386.pub2 PMC6464750 25052054

[B52] Zhuang X. Arridge S. Hawkes D. J. Ourselin S. (2011). A nonrigid registration framework using spatially encoded mutual information and free-form deformations. *IEEE Trans. Med. Imaging* 30 1819–1828. 10.1109/TMI.2011.2150240 21550878

[B53] Zu C. Jie B. Liu M. Chen S. Shen D. Zhang D. (2016). Label-aligned multi-task feature learning for multimodal classification of Alzheimer’s disease and mild cognitive impairment. *Brain Imaging Behav.* 10 1148–1159. 10.1007/s11682-015-9480-7 26572145 PMC4868803

