# Supplementary material for: Diagnosis of Alzheimer’s Disease via Multi-Modality 3D Convolutional Neural Network
Source: Front Neurosci. 2019 May 31;13:509. doi: 10.3389/fnins.2019.00509 (PMC6555226; doi:10.3389/fnins.2019.00509)
Supplement: Supplementary file 1 [file Table_1.DOCX]

In this work, the datasets were randomly split into training sets, validation sets and testing sets according to the patient IDs to ensure that all subjects of the same patient only appear in one set. Finally, 70% of a dataset was used as the training set, 10% as the validation set, and 20% as the testing set by random sampling. **Table 1, 2** summarized the information in different sets grouped by diagnosis. From the table, we can find the distributions of Age, Gender and MMSE are similar. This proved our sampling method is rational.

**Table 1** Summary of the Segmented dataset in Training/Validation/Testing set.

| **Diagnosis** | **Set** | **Number** | **Age^1^** | **Gender(M/F)** | **MMSE** |
| --- | --- | --- | --- | --- | --- |
| AD | Training | 970 | 76.0±7.47 | 562/408 | 21.8±4.19 |
|  | Validation | 187 | 77.5±7.39 | 101/86 | 21.8±5.12 |
|  | Testing | 198 | 75.3±7.65 | 109/89 | 22.4±4.12 |
| CN | Training | 1030 | 75.8±5.59 | 552/478 | 29.0±1.20 |
|  | Validation | 213 | 76.3±6.26 | 111/102 | 29.0±1.24 |
|  | Testing | 263 | 76.6±6.23 | 113/150 | 29.1±1.18 |
| Total | Training | 2000 | 75.9±6.57 | 1114/886 | 25.5±4.72 |
|  | Validation | 400 | 76.9±6.83 | 212/188 | 25.6±5.10 |
|  | Testing | 461 | 76.0±6.90 | 222/239 | 26.3±4.37 |

**Table 2** Summary of the Paired dataset in Training/Validation/Testing set.

| **Diagnosis** | **Set** | **Number** | **Age** | **Gender(M/F)** | **MMSE** |
| --- | --- | --- | --- | --- | --- |
| AD | Training | 441 | 76.3±7.2 | 256/185 | 21.8±3.94 |
|  | Validation | 62 | 77.1±7.9 | 28/34 | 20.5±4.46 |
|  | Testing | 144 | 76.2±6.96 | 76/68 | 21.6±4.34 |
| CN | Training | 520 | 76.3±5.95 | 289/231 | 28.8±1.31 |
|  | Validation | 71 | 77.2±6.15 | 42/29 | 28.9±1.13 |
|  | Testing | 140 | 75.1±6.14 | 90/50 | 29±1.17 |
| pMCI | Training | 230 | 74.9±7.1 | 156/74 | 25.9±2.42 |
|  | Validation | 32 | 73.8±6.76 | 18/14 | 26.3±1.49 |
|  | Testing | 64 | 76±7.1 | 38/26 | 26.1±2.45 |
| sMCI | Training | 293 | 74.5±7.41 | 197/96 | 27.9±1.74 |
|  | Validation | 51 | 74.2±7.18 | 34/17 | 27.2±1.81 |
|  | Testing | 97 | 74.2±7.6 | 66/31 | 27.5±1.88 |
| Total | Training | 1484 | 75.7±6.86 | 898/586 | 26.1±3.92 |
|  | Validation | 216 | 76±7.13 | 122/94 | 25.8±4.31 |
|  | Testing | 445 | 75.4±6.9 | 270/175 | 25.9±4.21 |

^1^ The values are denoted as mean ± standard deviation
